# Is survival improved by the use of NIV and PEG in amyotrophic lateral sclerosis (ALS)? A post-mortem study of 80 ALS patients

**DOI:** 10.1371/journal.pone.0177555

**Published:** 2017-05-23

**Authors:** Christian Burkhardt, Christoph Neuwirth, Andreas Sommacal, Peter M. Andersen, Markus Weber

**Affiliations:** 1 ALS Clinic/Neuromuscular Diseases Unit, Kantonsspital St. Gallen, St. Gallen, Switzerland; 2 Department of Pathology, Kantonsspital St. Gallen, St. Gallen, Switzerland; 3 Institute of Pharmacology and Clinical Neuroscience, Umeå University, Umeå, Sweden; Rutgers University, UNITED STATES

## Abstract

**Background:**

Non-invasive ventilation (NIV) and percutaneous gastrostomy (PEG) are guideline-recommended interventions for symptom management in amyotrophic lateral sclerosis (ALS). Their effect on survival is controversial and the impact on causes of death is unknown.

**Objective:**

To investigate the effect of NIV and PEG on survival and causes of death in ALS patients.

**Methods:**

Eighty deceased ALS patients underwent a complete post mortem analysis for causes of death between 2003 and 2015. Forty-two of these patients consented for genetic testing. Effects of NIV and PEG on survival and causes of death were analyzed in a multivariable Cox proportional hazard regression.

**Results:**

Six patients, who requested assisted suicide causing drug-induced hypoxia, were excluded from final analysis. Respiratory failure was the main cause of death in 72 out of 74 patients. Fifteen out of 74 died of aspiration pneumonia 23/74 of bronchopneumonia and 8/74 of a combination of aspiration pneumonia and bronchopneumonia. Twenty died of hypoxia without concomitant infection, and six patients had pulmonary embolism alone or in combination with pneumonia. NIV (p = 0.01) and PEG (p<0.01) had a significant impact on survival. In patients using NIV bronchopneumonia was significantly more frequent (p <0.04) compared to non-NIV patients. This effect was even more pronounced in limb onset patients (p<0.002). Patients with *C9orf72* hexanucleotide repeat expansions showed faster disease progression and shorter survival (p = 0.01).

**Conclusion:**

The use of NIV and PEG prolongs survival in ALS. This study supports current AAN and EFNS guidelines which recommend NIV and PEG as a treatment option in ALS. The risk of bronchopneumonia as cause of death may be increased by NIV.

## Introduction

Death is still the definite hallmark and a major primary endpoint in many treatment studies in amyotrophic lateral sclerosis (ALS), because reliable biomarkers to measure therapeutic effects are lacking.

Several epidemiological, but only few autopsy studies, have demonstrated that the main cause of death in ALS is respiratory failure. Other causes of death in ALS are pulmonary embolism, asphyxia, cardiac arrest or suicide.[1–3] An earlier study suggested that defining the cause of death based only on clinical observations is unreliable to determine the actual cause of death in ALS patients.[3] Even if clinical and autopsy records are available a relevant proportion of deaths cannot be determined or are classified as “sudden death”.[2,3] Moreover with respect to respiratory failure a clear distinction between bronchopneumonia, aspiration pneumonia and hypoxia becomes, with increasing post-mortem interval more and more difficult, as tissue degradation begins immediately after death.

In 1999 the first evidence-based guidelines for the clinical care of ALS patients and their relatives of the American Academy of Neurology (AAN)[4] were published. Their revision[5] and the European Federation of Neurological Societies (EFNS) 2005[6] and 2012[7] guidelines recommend multidisciplinary care and symptom management including the use of NIV and PEG to above all maintain quality of life for, but also to extend the life of patients with ALS. A controlled study demonstrated that NIV prolongs survival in patients without severe bulbar dysfunction,[8] but it has been difficult to show if the use of PEG affects survival.[9] Due to the lack of randomized controlled trials, it is still controversial if PEG feeding can positively influence survival or prevent weight loss.[9,10] It is also unknown whether NIV or PEG might have an influence on the cause of death in ALS patients. To elucidate if and how these well-established treatments in ALS care affect disease duration and causes of death we here assessed the effects of these interventions in the largest ALS autopsy cohorts after publication of the guidelines to date.

## Methods

This retrospective study was approved by the Ethics Committee of the Canton of St. Gallen in accordance with the ethical standards of the Helsinki Declaration.[11] Written informed consent was obtained from all patients and discussed as part of advance directives. The study adhered to Swiss legislations, regulations and guidelines regarding terminal care, end-of-life decisions by the patient and human autopsies. Forty-two of these patients also consented in writing to participate in genetic research and had DNA analysis performed for a panel of ALS genes (details of the genetic data are available upon request from the corresponding author: chris.burkhardt@gmx.de or Markus Weber: markus.weber@kssg.ch). C9orf72-mutations screening was performed using RPPCR coupled with amplicon length analysis, and in positive cases confirmed by Sountern blot” as described. [12] The clinical data was gathered during regular visits every three months in our outpatient clinic at the specialized ALS Clinic at the Kantonsspital St. Gallen from 2003 to 2015 and on reports from 80 consecutive collected autopsies of ALS patients. Death was confirmed by an independent physician. Autopsies were performed by the Department of Pathology of the Kantonsspital St. Gallen. The diagnosis of ALS was established according to the revised El Escorial criteria[13] and confirmed in all cases by autopsy. Demographic data included gender, time and site of symptom onset (defined as paresis), time from onset to diagnosis (diagnostic delay), time of death, time from death to autopsy, body mass index (BMI) before or at time of diagnosis and at death. In addition the revised amyotrophic lateral sclerosis functional rating scale (ALSFRS-R)[14], time of initiation of NIV or insertion of PEG, information on concomitant diseases and intake of Riluzole were recorded. Survival time was defined as time from symptom onset to death. Diagnostic delay was defined as time from symptom onset to diagnosis. The indication for initiation of NIV was, as recommended by the guidelines based on clinical signs of respiratory insufficiency in combination with respiratory parameters (forced vital capacity and sniff nasal pressure) with subsequent, nocturnal polysomnography [5,7]. Recommendation for PEG was given, if progressive malnutrition (weight loss >10%) or risk of aspiration was evident despite of a multidisciplinary nutrition management [5,7]. The respiratory status was not taken into account for the decision of PEG as all patients received NIV during PEG insertion to reduce the risk of hypoxia [15].

Assisted suicide by a physician is not explicitly regulated by Swiss law. But assisted suicide in a person that has the decisional capacity and the person assisting–physician or lay person–is not motivated by reasons of self-interest is tolerated.

Defining clinical causes of death were based on the patient history, clinical information and on macroscopic and if necessary microscopic analysis of the viscera. Different sections of the brain, spinal cord, peripheral nerves and various muscles were neuropathologically examined. Sections from 3 different spinal cord levels cervical (segment C6/7), thoracic (Th6) and lumbar (L3) were stained with hematoxylin and eosin (HE) and immune histochemically stained for ubiquitin and glial fibrillary acidic protein (GFAP). Particular attention was paid to ALS-typical, intracytoplasmatic, neuropathological changes including the presence of Bunina bodies, skein-like inclusions and threads, motor neuron loss, astrocytosis and sclerosis of the pyramidal tracts to confirm the diagnosis of ALS. Tissue specimens of the heart, liver, lung, spleen, kidney, pancreas, adrenal gland and occasionally other organs were fixed in formalin and afterwards mold in paraffin blocks. The sections were stained with HE (and if necessary with additional staining’s such as Periodic acid–Schiff, Elastic Van Gieseon or iron staining) and histologically examined. Causes of death were categorized as: aspiration pneumonia, bronchopneumonia, hypoxia, pulmonary embolism, assisted suicide, cardiac ischemia, peritonitis or combined respiratory cause of death consisting of aspiration pneumonia and/or bronchopneumonia and/or hypoxia. Aspiration pneumonia was assumed if aspiration material was found macroscopically in the lungs. On histological examination aspiration pneumonia was diagnosed in the presence of putrid pneumonia intermixed with aspiration material, squamous epithelium (from the esophagus and oropharynx) and bacteria as well as characteristic brownish coagulation necrosis and hemorrhages of lung tissue as a reaction of gastric acid. A sudden acute fatal aspiration shows no vital reaction to gastric acid and aspiration material at the histological level. By contrast bronchopneumonia presents merely with putrid inflammation. Isolated respiratory insufficiency (hypoxia) as cause of death was diagnosed with proof of lung dystelectasis, increased amount of macrophages in the alveolar spaces and fresh hypoxic necrosis of hippocampal neurons.

### Statistical analysis

Demographic and clinical characteristics of the participants are reported as percentages for categorical variables and means (SD) and medians (IQR) for continuous variables. Statistical analysis was performed using the R programming language (version 3.1.0, https://www.r-project.org). A threshold of *p* < 0.05 (two-tailed) was considered significant. About half of ALS patients were unable to visit our outpatient clinic during the last months of their lifes. As applied previously,[16] the ALSFRS-R at the time of death was estimated using linear regression for each patient separately, based on three or more ALSFRS-R scores obtained at earlier visits. Negative predicted ALSFRS-R estimates (n = 4) were rounded up to 0. Effects of NIV and PEG on survival were adjusted in a Cox proportional hazard regression (Cox PH) for various factors (diagnostic delay, region of onset, predicted ALSFRS-R, gender and age at diagnosis, BMI loss). Riluzole was not included as a cofactor because more than 90% of the patients used it on regular basis. Patients, who committed assisted suicide and those with truncal onset, were not taken into account for the survival analysis to minimize the bias. For statistical analyses of causes of deaths the two patients, who died from peritonitis or from acute heart failure, patients who committed assisted suicide, as well as three patients with truncal-onset (presenting with respiratory insufficiency as first symptom) were also excluded. The impact of genetic mutations on survival was also computed with a multivariable Cox PH model adjusting for diagnostic delay, predicted ALSFRS-R, gender, age, NIV and PEG. A multinomial logistic regression was used to examine the effect of NIV and PEG use on cause of death, adjusting again diagnostic delay, onset region, predicted ALSFRS-R, BMI loss, sex and age at diagnosis.

## Results

Basic demographic and clinic data of the cohort are reported in Table 1.

**Table 1:** Characteristic of the autopsy cohort.

Characteristics	ALS (n = 80)			
**Age of onset (years)**				
	median	64.0		
	mean	62.6		
	SD	13.1		
	range	28–84		
**Gender, n (%)**				
	male	47 (60%)		
	female	32 (40%)		
**Site of onset**				
	limb	55 (69%)		
	truncal	3 (4%)		
	bulbar	22 (27%)		
**Duration of disease (months)**				
	median	36.5		
	mean	43.2		
	SD	27.1		
	range	7–159		
**Delay of diagnosis (months)**				
	median	8.7		
	mean	13.7		
	SD	13.4		
	range	1–77		
**BMI**		at diagnosis	vs	at death
	median	23.7	vs	20.2
	mean	24.3	vs	20.7
	SD	3.81	vs	3.95
	range	18.9–39.5	vs	12.1–32.3
**Weight loss in BMI (% of original body weight) during disease**				
	mean	3.8 (16.6%)		
	range	-8.0–10.9 (-37.6–46.2%)		
**Time from death to start of autopsy (hours)**				
	median	4.25		
	range	1.25–58		
**Predicted ALSFRS-R score at death (n = 62)**				
	median	16.7		
	mean	17.5		
	SD	10		
	range	0–38.9		
**Riluzole use**	73/80 (91.25%)			

Most common concomitant diseases are listed in S1 Table in the supplement. The predicted mean ALSFRS-R score at death was 16.7. The clinical diagnosis of ALS was neuro-pathologically confirmed in all cases. The median time from death to autopsy was 4 hours. In all cases the cause of death could be autoptically clarified. Mean diagnostic delay in bulbar onset patients was 6 months shorter than in spinal onset patients (9.7 months versus 15.7 months, p = 0.05). Shorter diagnostic delay was significantly associated with shorter survival (HR, 0.97; 95% CI, 0.95–0.99; p = 0.01). After adjustment for various factors bulbar onset patients had a non-significant shorter median survival compared to limb onset patients (32.7 months versus 46.3 months, HR, 0.53; 95% CI, 0.27–1.06; p = 0.07). Three-quarters of the patients died at home or in nursing homes. One quarter of the patients had to be hospitalized due to acute respiratory deterioration.

In nine (seven with positive family history) out of 42 cases a pathological repeat expansion in *C9orf72* were found. Six of these patients had cognitive and behavioral changes or later developed full-blown frontotemporal dementia (FTD). Patients with *C9orf72* repeat expansion had a median survival of 28 months (excluding one patient committing assisted suicide) compared to patients without known mutation with a survival of 47 months. Multivariable analysis showed an increased hazard ratio (HR) of 5.73 (95% CI 1.5–21.86; p = 0.01) for patients with *C9orf72* repeat expansion compared with patients without expansion.

The mean loss in BMI in all ALS patients was 3.68 during the course of the disease (S1 Fig). The subgroup with severe weight loss (loss of >20% of the initial BMI) consisted mostly of limb onset patients (19/25 cases). Within this group with severe weight loss were 6 out of the 9 patients with *C9orf72* repeat expansions.

### Causes of deaths

**Fig 1:**
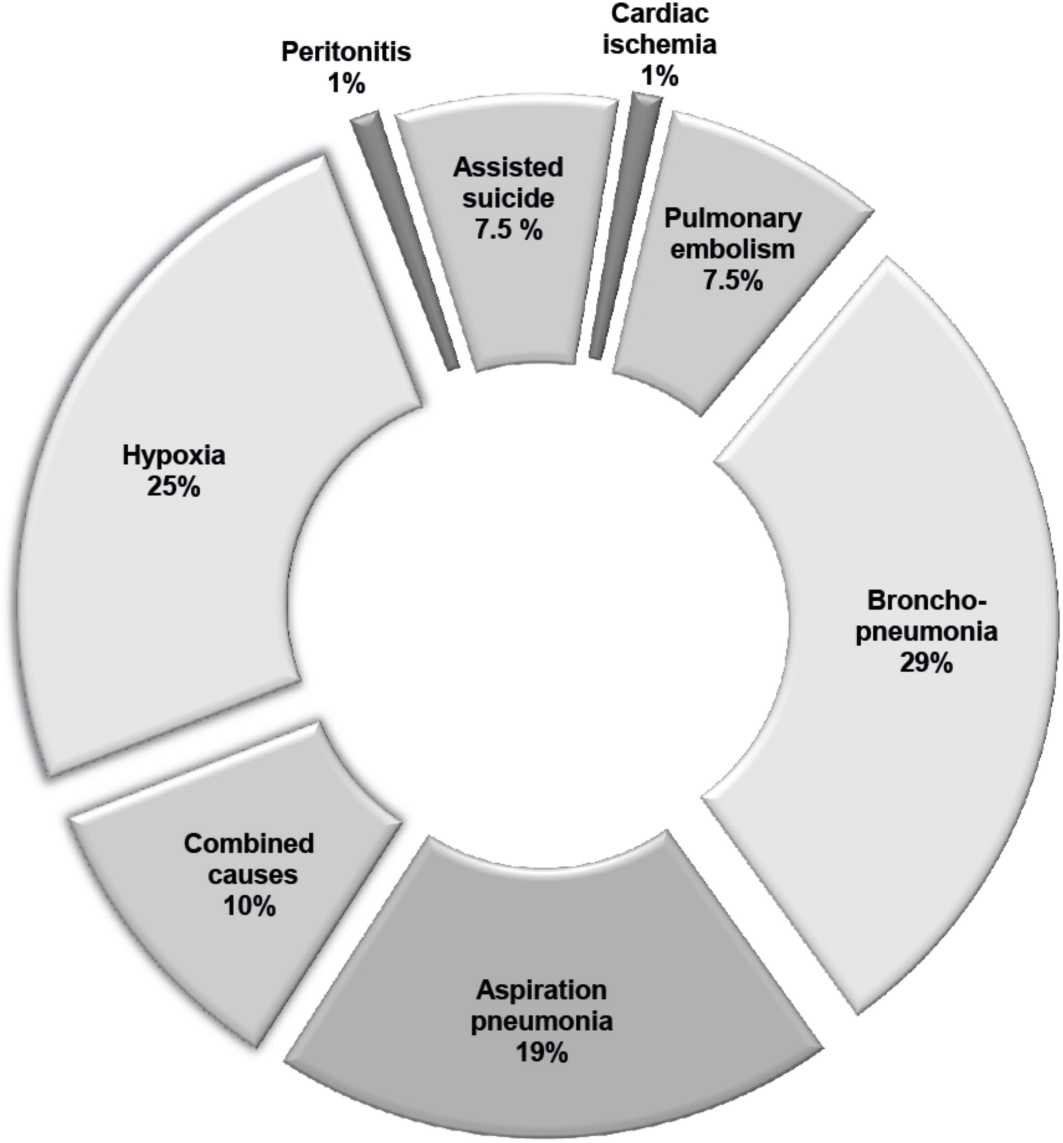
Autopsy confirmed causes of death in %.

Seventy-two out of 74 patients died of respiratory failure. Six patients died as a consequence of assisted suicide using Sodium-Pentobarbital. These Patients with assisted suicide had a longer median disease during (53.1 months) compared to the rest of cohort (36.3 months). NIV was used in only two of them and PEG in three patients. The reason for assisted suicide was diminished quality of life due to progressing loss of autonomy, diminished ability of communication or lack of will to live. One out of 74 patients died from spleen rupture with bleeding and peritonitis as a complication after PEG insertion during hospitalisation and one from cardiac ischemia. By far the most common cause of death was pneumonia (46/74) either caused by aspiration in 15/74 patients or by bronchopneumonia in 23/74 patients. Twenty out of eighty patients died from hypoxia. Twelve out of these 20 patients received NIV. Eight patients showed a combined respiratory cause of death (Fig 1).

In six patients (7.5%) acute pulmonary embolism (PE) alone or in combination with pneumonia led to death. Within this group of pulmonary embolism in ALS the ratio between spinal and bulbar onset was balanced (3 leg vs. 3 bulbar onset patients). Four patients were still able to walk within the last weeks before death caused by PE and there was no significant difference in the predicted ALSFRS-R scale (p = 0.39) compared to patients with other causes of death. None of these patients had a prior history of deep venous thrombosis or any other embolic event or any known risk factor for deep vein thrombosis. Detailed information about causes of death in the different ALS phenotypes are listed in S2 Table in the supplement.

### Effects of NIV

Thirty-eight out of 71 patients received NIV during the course of the disease (Table 2).

**Table 2:** Impact of NIV and PEG on survival*.

**Characteristics**	**NIV -**	**NIV +**	**PEG -**	**PEG +**
**Number of patients**	33/71	38/71	25/71	46/71
**Age at onset (years)**	64.4	61.3	64.1	61.9
**Region of onset**				
• limb onset	26/33	25/38	22/25	31/46
• bulbar onset	7/33	13/38	3/25	25/46
**Median time to initiation (months)**				
• overall		35.3		25.9
• limb onset		41.8		36.6
• bulbar onset		23.2		19.6
**Median survival (months)**				
• overall	36.5	47.7	29.8	40.1
• limb onset	39.5	54.3	30.2	52.3
• bulbar onset	24.2	39.7	14.1	32.7
**Cox proportional hazard regression model on survival (overall):**	**for NIV**		**for PEG**	
HR (95% CI)	0.19	(0.05–0.67) (p = 0.01)	0.24	(0.09–0.62) (p<0.01)

Abbreviations: HR, hazard ratio, CI, confidence interval

*patients who committed assisted suicide and truncal onset patients were excluded for survival analysis.

Median disease duration from symptom onset to death was more than 10 months longer in patients receiving NIV compared to patients without NIV (47.7 months versus 36.5 months). Univariate Cox-regression revealed no significant survival benefit for the use of NIV, but after adjusting for various cofactors (see methods) NIV analysis significantly increased survival across the whole cohort (HR, 0.19; 95% CI, 0.05–0.67; p = 0.01).

Subgroup analysis of limb onset (HR, 0.75; 95% CI, 0.34–1.64; p = 0.47) and bulbar (HR, 0.72 95% CI, 0.14–3.72; p = 0.70) patients even showed a greater survival benefit of almost 15 months in NIV users. This difference did not reach statistical significance, probably due to the small sample size in each group. When comparing this survival benefit between limb and bulbar onset patients using NIV there was no significant difference (p = 0.07) (Table 2).

**Fig 2:**
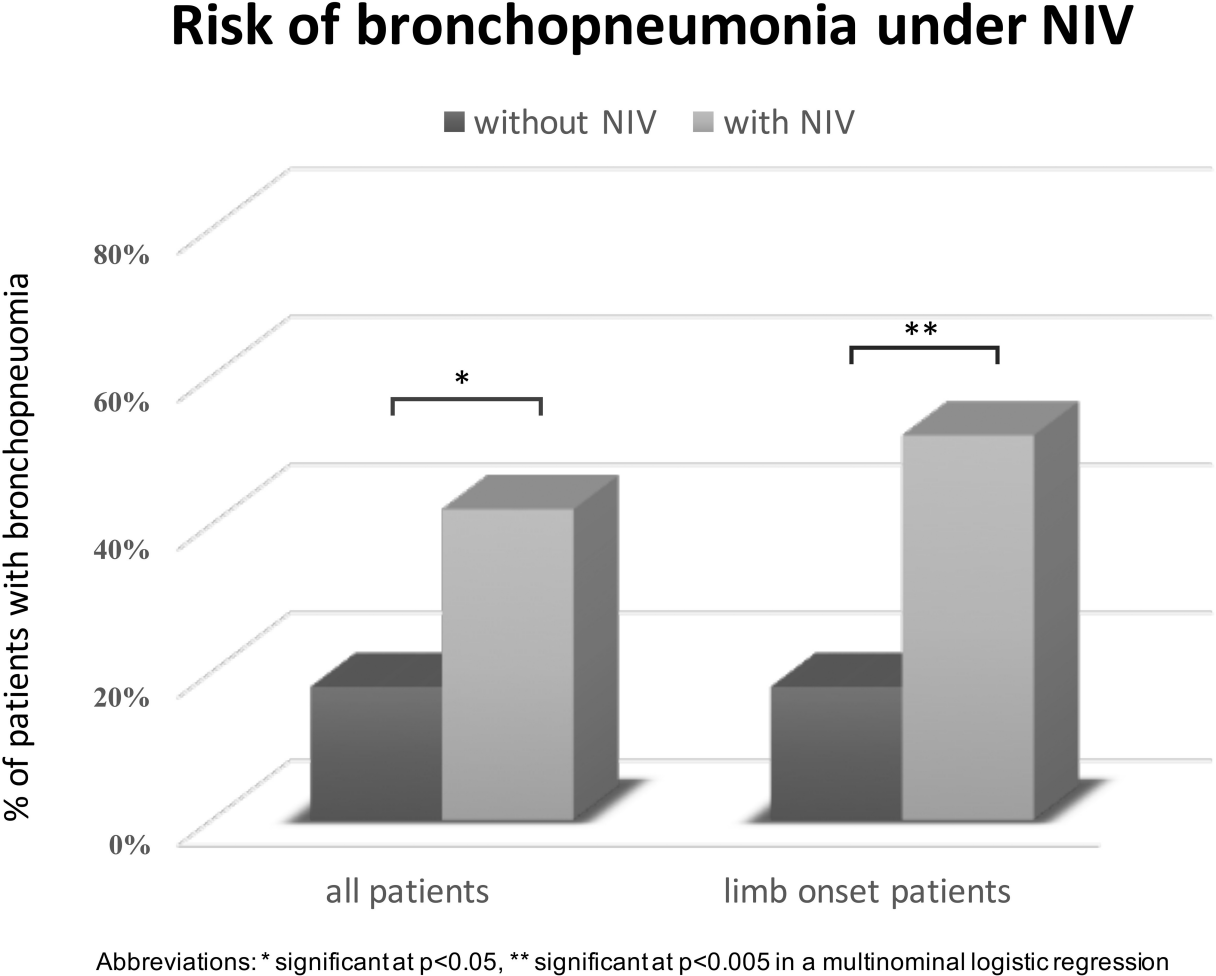
Risk of bronchopneumonia under NIV.

Bronchopneumonia occurred, as the only cause of death, more frequently (OR, 3.657; 95% CI 1.27–11.78, p = 0.021) in NIV (17/41) compared to non-NIV users (6/33). This difference was more pronounced in the subgroup of limb onset patients (Fig 2).

In this subgroup 13 out of 25 of these cases could be attributed to bronchopneumonia as cause of death compared to a small proportion of bronchopneumonia in patients not using NIV (3/26) (OR, 12.16; 95% CI 2.81–74.05; p<0.002).

### Effects of PEG

PEG was inserted in 46 patients. Comparing groups with and without PEG feeding (29.8 months versus 40.1 months) with univariate Cox regression revealed no significant positive effect on survival. After adjustment for various cofactors (see Methods) PEG insertion revealed a significant survival benefit (HR, 0.24; 95% CI, 0.09–0.62; p<0.01, Table 2).

BMI at death and BMI loss during the disease course did not differ between the group with PEG (BMI 23.8, -16.8%) and without PEG (BMI 23.8, -17.4%). Noticeably, causes of death did not significantly differ between patients with and without PEG. Patient treated with the combination of PEG and NIV showed a non-significant trend for longer survival compared to NIV-only (HR, 0.34; 95% CI 0.10–1.14; p = 0.08) or to PEG-only treated patients (HR, 0.51; 95% CI 0.21–1.26; p = 0.15).

## Discussion and conclusion

This post-mortem study comprises 80 ALS patients and is unique as a very short delay until autopsy (median 4 hours) allowed a clear definition of causes of death. It is the only autopsy- supported study after establishment of the AAN[4,5] and EFNS[6,7] guidelines. Treatment adhered to these guidelines especially with regard to NIV and PEG implementation. Despite the diagnostic advances over the last decades in other neurodegenerative diseases such as dementia and extrapyramidal syndromes, in at least 10 to 25% of cases, the final diagnosis can only be clarified by autopsy.[17–19] By contrast diagnostic certainty in ALS is autopsy-proven excellent, if the current used clinical diagnostic criteria are adhered to stringently.[2,3]

In this post-mortem study we confirm that respiratory failure is the main cause of death in almost all ALS patients (72/74).

In epidemiological studies respiratory dysfunction has been accountable for death in ALS up to 80%.[20,21] The proportion of pneumonia in these studies has been estimated around 15%.[22, 23] By contrast pneumonias stand out as the leading cause of death in 58% in our study, most often as bronchopneumonia (29%), less frequent aspiration pneumonia (19%) or a combination of both with or without hypoxia (10%). Our findings correlate well with the previous autopsy studies with an incidence of pneumonia in 55%[2] and 71%,[3] respectively. The incidence of pneumonia in general, but in particular silent aspiration pneumonia seems to be widely underestimated, if just based on clinical judgement. Until today only few studies dealt with the problem of aspiration in ALS with a wide range of assumed aspiration from 13% in epidemiology-based studies to 90% in a fiber-optic endoscopy evaluation.[24–26] This study shows that about 1/5 patients died of autopsy-verified aspiration pneumonia, which is a higher proportion as compared to previously published studies (16% and 11% respectively).[2,3]

These studies however did not provide information about post-mortem interval until autopsy and clear definitions of bronchopneumonia, aspiration pneumonia and hypoxia. The lower rate of aspiration pneumonia as well as the high proportion of unknown causes of death of up to 8% in these autopsy studies may be attributed to a longer postmortem interval, as progressive tissue degradation complicates the clear allocation of the cause of death.

In NIV patients survival was about 10 months longer compared to the group of non-NIV patients. This survival benefit was even more pronounced in the subgroups limb and bulbar onset patients, but did not reach statistical significance. The loss of significance despite the larger effect on survival can most likely be attributed to the too small sample size. However, this observation is in line with a recent observational study showing that also bulbar onset patients have a significant survival benefit by using NIV.[27]

Our results show that PEG insertion is a safe procedure.[15] Seventy-five percent of patients with PEG lived 6 months or more after insertion and only one out of 46 patients had a severe complication. Survival was, after adjustment for cofactors, significantly extended by the use of PEG feeding. This survival benefit can however not be explained by mean BMI at death or weight loss from symptom onset to death since these parameters were not influenced by PEG insertion. Whether prevention of aspiration pneumonia can account for the survival benefit remains unclear as this study revealed no significant difference. The positive effect of NIV and PEG on survival leads to the hypothesis that their combination might be superior to each treatment alone. An unexplained finding is that patients using NIV died significantly more often of bronchopneumonia as compared to non-NIV users. This effect was more significant in the subgroup of limb onset patients. Possible explanations are that longer survival exposes patients to a higher risk for pulmonary infection or alternatively NIV itself may be a risk factor for bronchopneumonia. While invasive ventilation is an accepted risk factor for pneumonia, especially for nosocomial infections,[28] NIV has been used to reduce the risk of nosocomial pneumonia on the ICU[29] and to treat patients with acute pneumonia.[30]

However, until now there are no studies addressing, whether chronic NIV use could by itself increase the risk for pneumonia in neuromuscular patients.

The rate of pulmonary embolism (7.5%) in this study was comparable with observation of a previous post-mortem study,[3] reporting pulmonary embolism in 6%. Epidemiological investigations in elderly non-ALS subjects estimate the annual risk of PE in the age group 65–69 years with 1.3 per 1000, steadily increasing with age to 2.8 in the age group 85–89 years.[31] Our data therefore imply a 2–3 fold increased risk for PE in ALS compared to these groups. Recently a prospective study[32] suggested that patients with leg weakness and reduced mobility carry a high risk of developing thromboembolic events. By contrast in our series 4 out of 6 patients with pulmonary embolism were still able to walk within the last weeks of their lives and the ALFFRS-R was not significantly different compared to the patients not dying from embolism. All but one patient with embolism were over 74 years suggesting that age may be a relevant risk factor for PE in ALS patients.

Limitations of this study include a possible selection bias from a tertiary ALS center with a selected patient group and missing data, especially on objective respiratory parameters such as blood gas values, FVC and SNIP to assess the respiratory function adequately before death, the adherence to NIV and PEG or about the use of cough assisting devices. These data could possibly give an indication whether NIV is a true risk factor for bronchopneumonia.

In summary, this study provides post mortem-proven evidence that adherence to current guidelines with respect to NIV and PEG significantly prolongs survival. This beneficial effect of NIV seems also to be present in bulbar onset patients. Patients receiving NIV, especially with limb onset, may be at higher risk to die from bronchopneumonia. Consequently an optimized prophylactic pneumonia management and early empiric antibiotic treatment should be considered in ALS patients in patients using NIV.

## Supporting information

S1 Table Concomitant disease found during autopsy. Abbreviations: Absolute number of cases with concomitant diseases not leading to death in the series of 80 autopsies.
(PDF)

S2 Table Causes of death for different ALS phenotypes. Abbreviations: between the brackets are the absolute numbers of ALS phenotypes or causes of death displayed.
(PDF)

S1 Fig Weight loss during the course of ALS. Abbreviations: Weight loss between symptom onset and death (in % of the initial body weight at symptom onset). Between the brackets are the absolute numbers of patientes in each group listed.
(PDF)
