# Supplementary material for: Is survival improved by the use of NIV and PEG in amyotrophic lateral sclerosis (ALS)? A post-mortem study of 80 ALS patients
Source: PLoS One. 2017 May 23;12(5):e0177555. doi: 10.1371/journal.pone.0177555 (PMC5441602; doi:10.1371/journal.pone.0177555)
Supplement: S1 Table — Abbreviations: Absolute number of cases with concomitant diseases not leading to death in the series of 80 autopsies. (PDF) [file pone.0177555.s001.pdf]

S1 Table: Concomitant disease found during autopsy

**Most common concomitant disease**	
**Vascular diseases**	
• coronary heart disease, atherosclerosis	**52/80**
• valvular heart disease	
• history of stroke/cardia ischemia	
**Thrombosis/pulmonary embolisms (prior history)**	**5/80**
**Pulmonary diseases**	
• emphysema	**22/80**
• chronic obstructive pulmonary disease (COPD)	
• obstructive sleep apnoe syndrome (OSAS)	
**Diverticulosis**	**12/80**
**Neoplasm**	**12/80**
• incidental neoplasm during autopsy	3/80
• history of neoplasm (in remission)	9/80

Abbreviations: Absolute number of cases with concomitant diseases not leading to death in the series of 80 autopsies
