# Supplementary material for: Is survival improved by the use of NIV and PEG in amyotrophic lateral sclerosis (ALS)? A post-mortem study of 80 ALS patients
Source: PLoS One. 2017 May 23;12(5):e0177555. doi: 10.1371/journal.pone.0177555 (PMC5441602; doi:10.1371/journal.pone.0177555)
Supplement: S2 Table — Abbreviations: between the brackets are the absolute numbers of ALS phenotypes or causes of death displayed. (PDF) [file pone.0177555.s002.pdf]

S2 Table: Causes of death for different ALS phenotypes

Causes of death (n=80)	Clinical phenotypes of ALS (n=80)		
	spinal (55)	bulbar (22)	truncal (3)
**Bronchopneumonia (23)**	16	7	-
**Aspiration pneumonia (15)**	10	4	1
**Hypoxia (20)**	16	2	2
**Assisted suicide (6)**	4	2	-
**Pulmonary embolism (6)**	3	3	-
**Combined causes of death (8)**	5	3	-
**Peritonitis (1)**	-	1	-
**Cardiac ischemia (1)**	1	-	-

Abbreviations: between the brackets are the absolute numbers of ALS phenotypes or causes of death displayed.
