# Supplementary figures and images for: Is survival improved by the use of NIV and PEG in amyotrophic lateral sclerosis (ALS)? A post-mortem study of 80 ALS patients
Source: PLoS One. 2017 May 23;12(5):e0177555. doi: 10.1371/journal.pone.0177555 (PMC5441602; doi:10.1371/journal.pone.0177555)

# S1 Fig: Weight loss during the course of ALS in % of the initial BMI

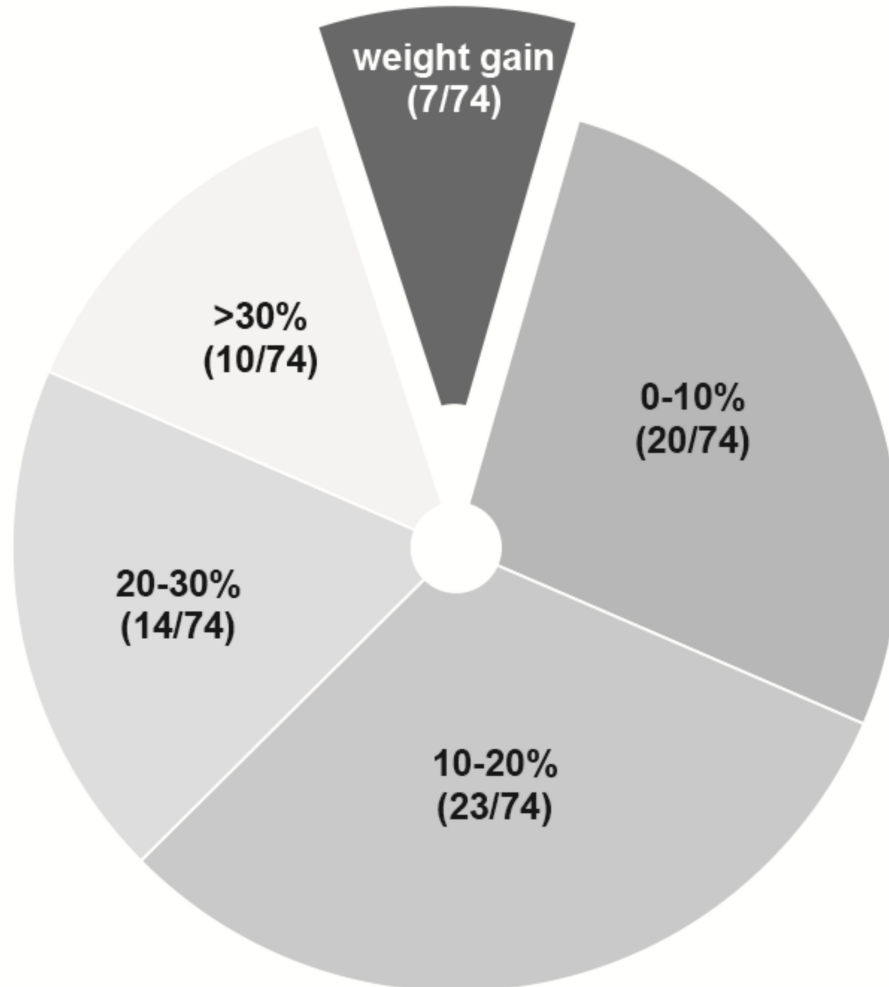

Supplement: S1 Fig — Abbreviations: Weight loss between symptom onset and death (in % of the initial body weight at symptom onset). Between the brackets are the absolute numbers of patientes in each group listed. (PDF) [file pone.0177555.s003.pdf]
